# Protoplasts: a friendly tool to study aluminum toxicity and coffee cell viability

**DOI:** 10.1186/s40064-016-3140-2

**Published:** 2016-08-30

**Authors:** Wilberth Poot-Poot, Beatriz A. Rodas-Junco, J. Armando Muñoz-Sánchez, S. M. Teresa Hernández-Sotomayor

**Affiliations:** 1 Unidad de Bioquímica y Biología Molecular de Plantas, Centro de Investigación Científica de Yucatán (CICY), Calle 43 No. 130, Col. Chuburná de Hidalgo, 97205 Mérida, YUC Mexico; 2 CONACYT, Facultad de Ingeniería Química, Campus de Ciencias Exactas e Ingeniería, Universidad Autónoma de Yucatán, Periférico Norte, Km 33.5, Tablaje catastral 13615 Col Chuburná de Hidalgo, 97203 Mérida, YUC Mexico

**Keywords:** Aluminum toxicity, *C. arabica*, DNA damage, Morin, Protoplasts

## Abstract

**Objective:**

Aluminum toxicity is a major limiting factor with regard to crop production and quality in most acidic soils around the world. We propose the use of *C. arabica* L. protoplasts to evaluate the toxic effects of aluminum, the nuclear localization of aluminum and propensity of aluminum to cause DNA damage.

**Results:**

After protoplasts were exposed to aluminum (Al) for varying periods of time (0, 5, 10, 20 and 30 min), we detected a reduction in protoplast viability. Additionally, we observed a rapid decline in the ability of protoplasts to synthesize DNA following exposure to Al for 30 min. Furthermore, DNA damage was observed after 10 min of treatment with Al.

**Conclusions:**

Protoplasts can be used to evaluate the effects of Al upon entry into the cell, which affects the structure of the nucleus. These results indicate that protoplasts provide a useful model for the study Al toxicity at the cellular level.

## Background

Aluminum (Al) toxicity is primarily found in acidic environments. Therefore, Al toxicity is a major limiting factor of crop production and quality in many acidic soils worldwide. The phytotoxicity of Al has long been a subject of physiological and biochemical interest; Al toxicity has not yet been fully characterized due to the complicated chemistry of Al and the presence of the cell wall. Plant species have evolved diverse mechanisms of Al tolerance, including the secretion of Al-induced organic acids, the immobilization of Al in the cell wall, and increasing the pH of the rhizosphere (He et al. 2012). Al accumulates primarily in the cell wall; only a small fraction of Al interacts with membranes. It has been suggested that alterations can occur, which are reflected in reduced root growth. These changes include incremental increases in cell wall rigidity, the induction of callose synthesis, an alteration in the lipid composition of the plasma membrane, the disturbance of ion transport and Ca^2+^ homeostasis, rearrangement and alterations of the cytoskeleton, the production of toxic oxygen reactive species, and interactions between Al and double-stranded DNA (Gupta et al. 2013; Zheng and Yang 2005; Tabuchi and Matsumoto 2001).

Among the strategies employed against aluminum toxicity, the exudation of organic acids plays an important role in reducing the toxic effects of aluminum in the apoplast and symplast of roots and shoots (Klug and Horst 2010; Ma et al. 1997). Also a wheat gene encoding an aluminum-activated malate transporter ALMT1 (aluminum-activated malate transporter) has been described, and show that may increase tolerance to Al treatment (Sasaki et al. 2004, 2010). Symplastic detoxification, compartmentation, and translocation are the most important Al tolerance-mediating processes in Al-accumulating plant species (Gupta et al. 2013). However, these processes require Al transport through the cell wall and the plasma membrane (PM). Currently, our knowledge concerning the passage of Al through biological membranes is limited. Al^3+^ has a very strong affinity for the PM because of the negativity of the carboxyl and phosphate groups in the cell membrane (Ahn and Matsumoto 2006; Zheng and Yang 2005). Accumulating evidence suggests that the PM of cells at the root apex could be a primary site of Al toxicity (Gupta et al. 2013; Sónia 2012). It was reported that Al could alter the structure and function of the PM by interacting with lipids and causing lipid peroxidation (Ishikawa et al. 2001) or modifying the uptake of cations such as Ca^2+^, Mg^2+^, K^+^, or NH_4_^+^ (Gupta et al. 2013; Mariano and Keltjens 2005).

*Coffea arabica* is a woody species belonging to the genus *Coffea* of the family Rubiaceae, which grows in acidic soils. *C. arabica* cultivars represent a very important commercial crop worldwide. This crop grows in acidic soils where Al is present at micromolar concentrations and is released into the soil solution, which affects the growth and productivity of this cultivar (Ramírez-Benítez et al. 2008; Kochian et al. 2005). Even though we know that Al^3+^ toxicity decreases crop growth in acidic soils (Rao et al. 2016), the precise mechanism of Al^3+^ toxicity remains poorly understood.

The use of protoplasts from the organs and tissues of higher plants has made the comprehensive study of metal toxicity possible (Wagatsuma et al. 1995; Du and Bao 2005; Mizuhiro et al. 2001; Young-Sang et al. 2001). The use of protoplasts from cell suspensions allows for the close examination of the direct interaction between Al^3+^ and the plasma membrane (PM) and the movement of aluminum into the cell. These types of studies also avoid complications that might arise due to interactions between Al^3+^ and cell wall components. Ramírez-Benítez et al. (2009) found that after applying a 100 μM AlCl_3_ treatment to coffee protoplasts for short periods of time, aluminum passed through the plasma membrane.

Encouraged by previous reports, we proposed the use of protoplasts (Poot-Poot and Hernandez-Sotomayor 2011; Rueda et al. 2011; Panda and Matsumoto 2007; Marquès et al. 2004; Davey et al. 2005) as a practical model to study intimately the effect of metals such as Al on cellular processes. The aim of this work was to elucidate the mechanism by which aluminum confers toxic effects in crop plant cultures at the cellular level using isolated protoplasts from a *C. arabica* L. cell suspension; our research focused on nuclear interactions and DNA damage.

## Methods

### Plant material

Suspension cell culture of *Coffea arabica* var. Catuai was obtained from dispersed embryogenic callus and maintained in medium (Murashige and Skoog 1962) supplemented with 87 mM sucrose, 140 µM cysteine-HCl, 560 µM myo-inositol, 30 µM thiamine/HCl, 13.6 µM 2,4-dichloro-phenoxyacetic acid and 4.4 µM 6-benzyl-aminopurine (6-BAP) at pH 5.8 (Martinez-Estevez et al. 2001). Cell suspensions were cultured in the dark at 25 °C and shaken at 100 rpm and after 14 days in these conditions, fresh media was added to maintain the culture.

### Chemicals

Leupeptine, driselase, pectinase and morin reagent (2′, 3′, 4′, 5′, 7′-pentahydroxyflavone) were purchased from Sigma-Aldrich (St. Louis, MO, USA). Cellulase and macerase were purchased from Calbiochem (CA, USA), and rifampicin was purchased from Sigma-Aldrich (St. Louis, MO, USA).

### Isolation of protoplasts

The protoplasts were isolated from 16-day old cultures. Cells (1 g fresh weight) were digested with 10 ml of lysis solution (LS), which contained the following: sorbitol (400 mM), CaCl_2_ (6 mM), KCl (123 mM), sucrose (87 mM), NH_4_NO_3_ (10 mM) and KH_2_PO_4_ (625 µM) at pH 4.3 with hydrolytic enzymes (1 KU driselase g^−1^, 20 KU cellulase g^−1^, 6 KU macerase g^−1^, and pectinase 3.8 KU g^−1^). The LS was sterilized by filtration using Millex syringe-driven filter units (Millipore Corp., USA) with a 0.22 µm pore size. The mixture was later incubated on a rotator shaker (50 rpm) for 12 h in the dark at 28 ± 2 °C for protoplast isolation (Ramírez-Benítez et al. 2009). Rifampicin (4 mg l^−1^) and leupeptin (1 μg ml^−1^) were added to the LS prior to filtering. Then, protoplasts were separated from the cellular debris and enzymatic residue by centrifugation (200 rpm) for 3 min at 23 °C. Cells were washed three times with 5 ml of maintenance solution (MS; lysis solution without digestive enzymes). Finally, the protoplasts were transferred into tubes with 5 ml of MS for further treatment.

### Al treatment of protoplasts and visualization of Al^3+^ using morin

Protoplasts were isolated as described (4.4 × 10^6^ cells) and incubated for different periods of time (0, 5. 10, 20 or 30 min) in MS with 100 μM AlCl_3_. The incubation with AlCl_3_ was terminated by centrifuging the protoplasts at 50×*g* for 3 s. After the incubation, protoplasts were separated and observed using a fluorescence microscope and quantified with a hemocytometer camera. For Al^3+^ localization, the protoplasts were exposed to 50 μM of morin (Browne et al. 1990; Larsen et al. 1998) for 1 h before the treatment with 100 μM of AlCl_3_.

### Protoplast viability

The viability of the protoplasts was evaluated using the method described by Liu et al. (2008), which consisted of mixing 25 μl of a solution of fluorescein diacetate (FDA) (5 mg acetone ml^−1^) with 2 ml of MS. A 250 μl aliquot of the above mixture was added to 250 μl of a suspension of protoplasts (4.4 × 10^6^) that were untreated or treated with 100 μM AlCl_3_, as described above. The samples were incubated at room temperature for 15 min in the dark. The viability was determined by counting the green protoplasts with circular shapes under a fluorescence microscope (Axioplan, Zeiss) at 420–490 nm. Viability was expressed as the number of protoplasts that fluoresced yellow-green under illumination out of the total number of isolated protoplasts; the number of protoplasts in the sample without AlCl_3_ treatment (C) represented 100 % viability.

### Isolation of nuclei and observation of Al internalization

Morin is a fluorescent histochemical indicator of Al^3+^ (Browne et al. 1990; Larsen et al. 1998). The nuclei of untreated protoplasts and protoplasts treated with 100 μM AlCl_3_ were isolated using the method described by Saxena et al. (1985), with some modifications. The samples were centrifuged at 70×*g* for 5 min and resuspended in 600 μl of a nuclear isolation buffer (NIB) containing the following: 10 mM 2-(*N*-morpholino) ethanesulfonic acid (MES), 0.2 M sucrose, 0.1 % Triton X-100, 2.5 M EDTA, 2.5 mM dithiothreitol (DTT), 0.1 mM spermine, 10 mM NaC1 and 10 mM KC1. Protoplasts were incubated with NIB on ice for 7 min and then lysed using a 21 × 32 mm diameter syringe. The nuclei were collected by centrifugation at 500×*g* for 10 min and resuspended in 50 μl of NIB. After AlCl_3_ treatment, the nuclei were mixed with 10 μl of 5 mg/ml of DAPI (4,6-Diamidino-2-phenylindole dihydrochloride) for 20 min. DAPI is a fluorochrome with a high affinity for the DNA double helix. Finally, the nuclei were observed under an Axioplan II epifluorescence microscope (Carl Zeiss MicroImaging Inc., Thornwood, NY, USA) equipped with excitation and emission filters for morin (470–520 nm) and DAPI (358–461 nm).

### Incorporation of [^3^H]-thymidine into DNA

For this study, 4.4 × 10^6^ protoplasts in 1 ml of MS were incubated with 100 μM AlCl_3_ for different periods of time at 25 °C in the dark. The incorporation of thymidine into DNA was monitored as described by Minocha et al. (1992), with some modifications. After the protoplasts were treated with AlCl_3_, the MS was removed; then, 370 KBq of [^3^H]-thymidine (2.5 μM cold thymidine) was added. The protoplasts were incubated at 25 °C for 30 min. After this incubation, ice cold perchloric acid (at a final concentration of 10 %) was added; the protoplasts were then incubated on ice for 1 h. DNA isolation was performed by centrifuging the previous mixture at 50×*g* for 45 s. The pellets were solubilized in 0.5 ml 0.2 M NaOH solution with 0.1 % SDS at 37 °C for 1 h. The mixture was neutralized with 0.5 ml 2 M Tris/HCl (pH 7.5). Finally, the samples were quantified using 5 ml of scintillation fluid in a scintillation counter (Beckman LS-6500, Mexico). Each measurement was carried out using three independent experiments.

### Analysis of DNA damage in protoplasts

Protoplasts (1 × 10^6^) were treated 100 μM AlCl_3_ for different periods of time at 25 °C. Samples were centrifuged at 60×*g* for 7 min. Protoplasts (200 μl) were placed in tubes, and 18 μl of extraction solution (50 mM NaCl, 1 mM Na_2_EDTA, 0.5 % SDS, pH 8.3) was added for DNA extraction. The tubes were stored on ice for 30 min.

After this incubation, 3 μl of loading buffer (6X; 0.25 % bromophenol blue, 0.25 % xylene cyanol and 30 % glycerol) was added. The samples were loaded onto an agarose gel containing 1 % ethidium bromide. Finally, electrophoresis was performed at 60 volts for 1 h in 1X Tris/acetic acid/EDTA (TAE) buffer. The protoplast DNA from three independent experiments was analyzed for damage.

### Statistical analyses

The experiments were independently replicated in triplicate, and the data were analyzed by ANOVA using Statgraphics 5.1 software (Madrid, Spain) and means were compared by Student’s *t* test with a level of significance of *p* < 0.05.

## Results

### Effect of Al on protoplast cell viability

**Fig. 1:**
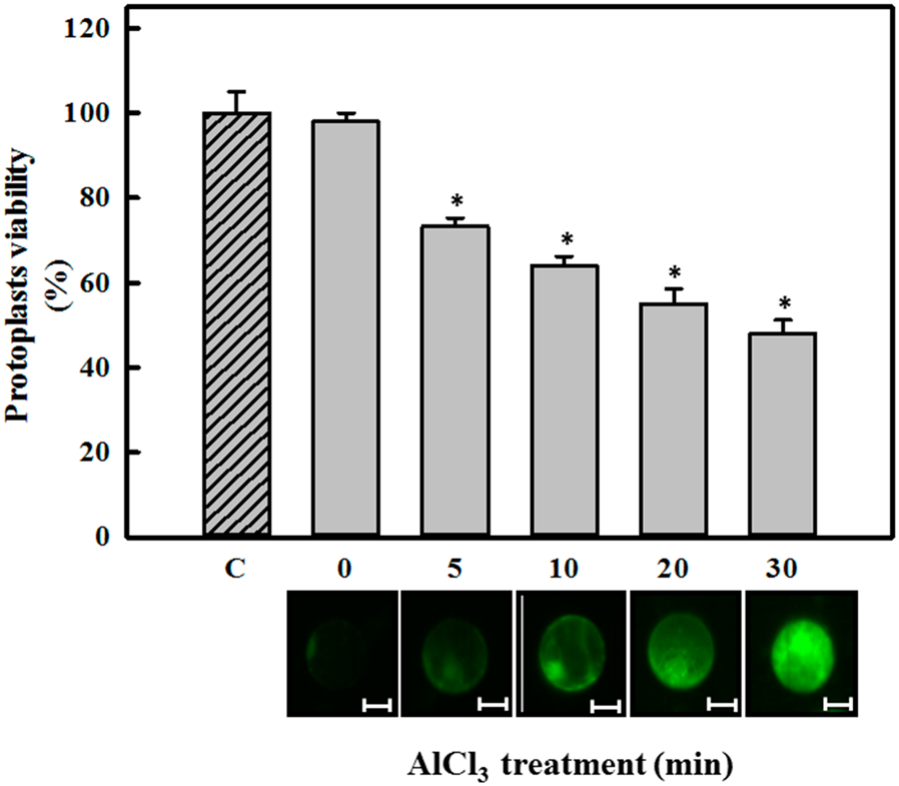
Effect of AlCl_3_ on the viability of protoplasts from *C. arabica* suspension cells (*top* figure). Protoplasts were treated with 100 μM AlCl_3_ for different periods of time. Protoplasts were then incubated with FDA, and the viability was determined as described in the “Methods” section. The *dashed bar* (*C*) shows the viability of protoplasts incubated for 30 min in the absence of AlCl_3_, which is representative of 100 % viability. Each *bar* represents the mean of three independent experiments ±SD. *Asterisk*, *p* < 0.05 compared to the control (*C*). Aluminum enters into *C. Arabica* protoplasts (*bottom* figure). Protoplasts were incubated with 50 µM morin for 1 h before AlCl_3_ treatment (100 µM) for different time periods (0, 5, 10, 20, or 30 min). *Green* fluorescence was emitted by Al:morin complexes. These results are representative of three separate experiments. *Bars* 40 μm

First, it was necessary to determine whether treating protoplasts with Al affects cell viability. As shown in Fig. 1 (top), an effect on protoplast viability was first observed after 5 min of treatment with AlCl_3_ (Fig. 1 top, gray bars). However, this effect was greater after 10 min of treatment with AlCl_3_; viability was reduced by up to 50 % compared to untreated protoplasts at 0 or 30 min (Fig. 1 top, dashed and zero bar).

### Aluminum enters into protoplasts

Al^3+^ was visualized using morin, as described in the “Methods” section, to determine whether Al entered into protoplasts. In this experiment, protoplasts were treated with 100 μM AlCl_3_ for 0, 5, 10, 20 or 30 min. The fluorescence signal (green color) emitted due to the morin-Al^3+^ interaction was observed via epifluorescence microscopy.

We have previously shown that Al was localized to a cellular zone similar to the nucleus (Ramírez-Benítez et al. 2009); this experiment was repeated to ensure that Al^3+^ penetrated into the cell (Fig. 1 bottom).

**Fig. 2:**
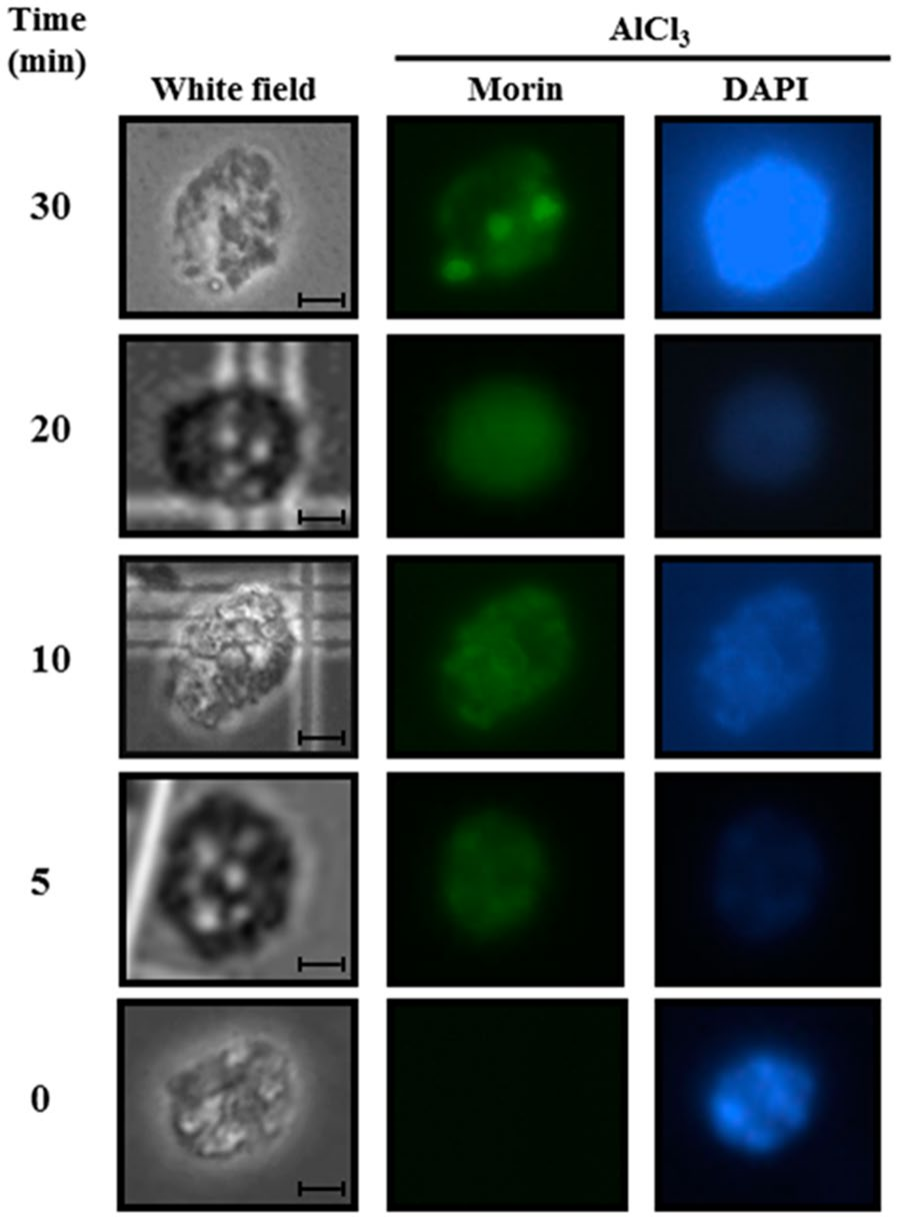
Presence of Al^3+^ in nuclei isolated from *C. arabica* protoplasts treated with AlCl_3_. Before treatment with 100 µM aluminum (0, 5, 10, 20 or 30 min), the protoplasts were exposed to 50 μM morin for 1 h. After exposure to morin and AlCl_3_, protoplasts were exposed to DAPI for 20 min. The nuclei were obtained as outlined in the “Methods” section. Morin and DAPI staining were observed at 470–520 and 365 nm, respectively, using a fluorescence microscope. *Bars* 40 μm

To complement these results, we isolated nuclei from protoplasts treated with 100 μM AlCl_3_ for different time periods to confirm that Al^3+^ was present in the nuclei. The nuclei had been previously stained with morin and DAPI, and the fluorescence signal was monitored using a microscope (Fig. 2).

A green signal was observed in protoplast nuclei after 5 min of treatment with AlCl_3_, as shown in Fig. 2 (middle panel). To verify the nuclear localization of the aluminum signal, a reagent specific for the nucleus (DAPI) was used. The presence of nuclei was confirmed in all samples at all time points. Nuclei were observed under a filter for morin (central column). The intensity of the green signal in all nuclear zones of cells increased when the incubation with AlCl_3_ increased from 5 to 30 min. In cells incubated for 30 min, the morin-aluminum signal shows that Al accumulated in three small zones of greater intensity compared to elsewhere in the nucleus. This result also indicates that aluminum enters the nucleus very quickly.

### Effect of Al on DNA synthesis

**Fig. 3:**
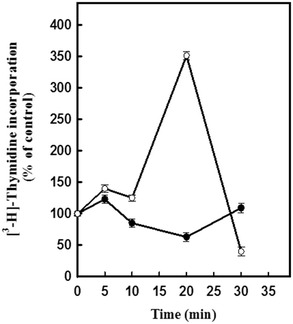
Incorporation of thymidine into the DNA of *C. arabica* protoplasts. Protoplasts were incubated with (*closed circle*) or without (*open circle*) 100 μM AlCl_3_ for different time periods (0, 5, 10, 20, or 30 min). [^3^H]-thymidine incorporation was determined as outlined in the “Methods” section. Results are the mean of three experiments ±SE and are expressed as a percentage of the [^3^H]-thymidine incorporated in the absence of AlCl_3_, which was considered to be 100 %

To evaluate the effect of Al on DNA synthesis in *C. arabica* protoplasts, we assayed the incorporation of thymidine into DNA. After protoplasts were treated with 100 μM AlCl_3_ for 0, 5, 10, 20 or 30 min, we observed that the incorporation of thymidine into DNA (Fig. 3, closed symbols) was less than the amount of ^3^H-thymidine incorporated into the DNA of untreated protoplasts (Fig. 3, open symbols). These results suggest an initial negative effect of aluminum on DNA synthesis in protoplasts.

### Detection of DNA fragmentation

**Fig. 4:**
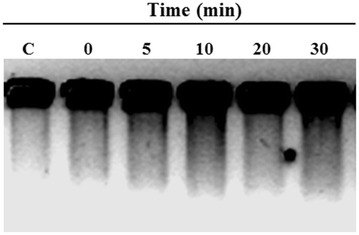
Analysis of protoplast DNA. Protoplasts were treated with 100 μM AlCl_3_ for different periods of time. After treatment, DNA was extracted as described in the “Methods” section. DNA was analyzed by electrophoresis on an agarose gel. These results are representative of three independent experiments. The sample in *lane C* represents DNA extracted from protoplasts incubated for 30 min without AlCl_3_

To investigate whether DNA fragmentation occurred after aluminum treatment, nuclear DNA from protoplasts was isolated and analyzed (Fig. 4). Protoplasts were obtained and treated with 100 μM AlCl_3_ for 0, 5, 10, 20 or 30 min. Following treatment, DNA was extracted and analyzed by gel electrophoresis. The results of these experiments are shown in Fig. 4.

The extent of DNA damage was represented by the length of the smear observed in the gel. At 0 min, no significant DNA degradation was observed. After only 5 min of treatment with AlCl_3_, a more intense signal indicating DNA degradation was present. Samples at 10, 20 and 30 min of treatment showed a longer smear, suggesting that aluminum possibly interacts with nuclear material and possibly induces DNA damage. This damage alters the response capacity of the plant cell.

## Discussion

Using morin as a marker of aluminum internalization, Ramírez-Benítez et al. (2009) observed the mobilization of aluminum (Al^3+^) into the cell. In this study, we used protoplasts as a user-friendly tool to study the effect of Al in plant cells. Our results demonstrate that short Al exposure times affected protoplast viability. We detected a negative effect of aluminum on protoplast viability (Fig. 1) up to 30 min after aluminum exposure; viability decreased to 52 % of the control (Fig. 1, bar C).

Previous studies have shown that different amounts of aluminum accumulate in the cell wall and the plasma membrane. Therefore, only a small amount of aluminum penetrates the plasma membrane and affects cytosolic targets (Gupta et al. 2013; Ahn and Matsumoto 2006; Zheng and Yang 2005). Other reports also indicate that aluminum can enter into plant cells. In root beans (Beta *vulgaris*) exposed to 50 μM aluminum, clear changes in cell viability and cell turgency were observed (in cells without a cell wall). These results are in agreement with those presented in Fig. 1.

This decrease in viability could be due to the interaction of aluminum with molecules such as enzymes (Liu et al. 2008; Ahn and Matsumoto 2006) or phospholipids (Gupta et al. 2013; Ahn and Matsumoto 2006), which would affect normal function. The capacity of phosphate and the carboxyl groups present in the plasma membrane to bind aluminum molecules has also been previously demonstrated (Gupta et al. 2013; Yamamoto et al. 2001); this binding can modify the transport properties of the membrane in protoplasts.

The reduction in cell viability may also be due to functional damage to organelles such as mitochondria. Mitochondria produce reactive oxygen species (ROS) and contribute to the oxidation of proteins, DNA (Siddiqui et al. 2013) and membrane phospholipids (Gupta et al. 2013; Ahn and Matsumoto 2006). Damage to mitochondria was observed in *Arabidopsis thaliana* protoplasts treated with 0.5 mM aluminum; increases in ROS and caspase-3 activity and a decrease in cellular viability (50 %) were observed after 60 min of treatment (Li and Xing 2010).

The outer surface of the PM seems to be a major target of aluminum. The binding of aluminum to membrane lipids results in the rigidification of the plasma membrane (Liu et al. 2014; Kochian et al. 2005). Several of the ways plant cells respond to aluminum seem to be related to alterations in PM function; these alterations include the blockage of Ca^2+^ channels, the depolarization of transmembrane electrical potential (Ahn and Matsumoto 2006; Rengel and Zhang 2003), the excretion of organic acids (Liu et al. 2014; Gupta et al. 2013), the peroxidation of lipids, and the inhibition of the H_2_O_2_-stimulated increase in IP_3_ (Ahn and Matsumoto 2006). Conversely, a significant effect on DNA synthesis (as measured by the incorporation of ^3^H-thymidine) was observed in protoplasts treated with AlCl_3_ compared with the control (Fig. 3). A rapid decline in the ability of the protoplasts to synthesize DNA in culture was also observed. These findings suggest that DNA synthesis in these protoplasts was affected by the presence of Al after a short exposure period (30 min). Also it is important to mention that the decline in thymidine incorporation can be explained by changes in the rate of thymidine incorporation into DNA. This may represent cycles of DNA duplication and the lack of DNA synthesis at specific points that reflect changes with the cell cycle machinery. Since the relative rate of DNA synthesis decreased with the age of cells, or as in this case protoplast, therefore the peak of DNA synthesis observed at 20 min may indicate a high level of synchrony during the first cycle followed by changes in the protoplast population in the fresh media.

Matsumoto et al. (1976) suggested the binding of Al to DNA inhibits cell division. The data presented in this study on the inhibitory effects of Al on DNA synthesis are consistent with observations by others. For example, Minocha et al. (1992) reported that treatment with aluminum resulted in the severe inhibition of DNA synthesis within 16–24 h.

We observed that the aluminum passes through the plasma membrane after 5 min; changes at the nuclear level, including changes in DNA integrity, become visible within 30 min of exposure (Figs. 2, 4). Changes were observed in nuclear structures (white arrows); these may be nucleoli or the initiation of the disintegration of nucleolar material to form structures such as those detected in *Allium cepa* L. cells (Fiskejo 1983, 1990). These Al-related structures should be investigated in subsequent studies to help elucidate the effects of aluminum on *Coffea arabica* L.

DNA fragmentation in plants has also been observed during germination (Wang et al. 1996) and following exposure to salt stress (Katsuhara and Kawasaki 1996) or aluminum (Surapu et al. 2014; Zhan et al. 2013). Al has a negative effect that arises from an increase in the rigidity of the DNA double helix; this can affect DNA composition and chromatin structure (Gupta et al. 2013). In this study, DNA damage was observed (Fig. 4); this damage possibly occurred due to direct interference by Al with ions such as Mg^2+^ or Ca^2+^ (Wallace and Anderson 1984). Katsuhara and Kawasaki (1996) reported that variations in cytoplasmic Ca^2+^ levels might activate certain endogenous Ca^2+^-dependent proteases to cleave chromatic DNA at the linker sites between nucleosomes; this cleavage results in the fragmentation of DNA and can originate from abiotic stress. Our study supports these findings; we observed DNA fragmentation under Al stress. Our results may be related to the previous findings of Surapu et al. (2014), who observed Al-induced DNA fragmentation in tomato seedlings.

## Conclusions

The results of this study show that Al has a deleterious effect on the viability of protoplasts and DNA synthesis and possibly affects DNA replication after short periods of time. We also present evidence of the presence of aluminum in the cytoplasm (Fig. 1 bottom) and in the nucleus (Fig. 2); additionally, the amount of Al in the nucleus seems to depend on the exposure time (Fig. 2). These results could indicate an unidentified process for the mobilization and accumulation of aluminum in the nucleus. The DNA degradation observed in protoplasts (Fig. 4) could lead to the inhibition of cell growth and cell death. It has been argued that DNA is a direct target of Al^3+^ in biological systems. Consequently, due to the predicted complexity of Al toxicity following internalization, it has been hard to envisage that singe changes in biochemical target such as DNA could result in a measurable increase in Al toxicity.

The mechanism underlying these Al toxicity phenomena need clarification; this could be provided by elucidating the mechanism of cell division blockage, the interrelationship between Al and the cell membrane, and the effect of Al on other organelles. This information is necessary to better understand the distribution of Al in the cell. Protoplasts are a user-friendly tool that can be leveraged to increase our knowledge of Al toxicity after short exposure times. The use of protoplasts is expected to contribute to a deeper understanding of these cellular responses, which will help us improve the performance of commercial crops that are susceptible to Al toxicity. In our laboratory, a stable cell line can be regenerated from protoplasts after different treatments, leading this o the possibility that the results obtained in this model can be extrapolated to cells with cell wall.

## Abbreviations

DAPI: 4,6-diamidino-2-phenylindole dihydrochloride
DTT: dithiothreitol
EDTA: ethylenediaminetetraacetic acid
FDA: fluorescein diacetate
IP_3_: inositol 1,4,5-triphosphate
MES: 2-(*N*-morpholino) ethanesulfonic acid
MS: Murashige and Skoog culture media
NIB: nuclear isolation buffer
PCA: perchloric acid
PM: plasma membrane
SDS: sodium dodecylsulfate
TAE: tris-acid acetic acid-EDTA
2,4-D: 2,4-dichloro-phenoxyacetic acid
rpm: revolution per minute

## References

[CR1] Ahn SJ, Matsumoto H (2006). The role of the plasma membrane in the response of plant roots to aluminum toxicity. Plant Signal Behav.

[CR2] Browne BA, Mcoll JG, Driscoll CT (1990). Aluminum speciation using morin: I. morin and its complexes with aluminum. Environ Qual.

[CR3] Davey MR, Anthony P, Powera JB, Lowe KC (2005). Plant protoplasts: status and biotechnological perspectives. Biotechnol Adv.

[CR4] Du L, Bao M (2005). Plant regeneration from protoplasts isolated from embryogenic suspension cultured cells of *Cinnamomum camphora* L.. Plant Cell Rep.

[CR5] Fiskejo G (1983). Nucleolar dissolution induced by aluminium in root cells of Allium. Plant Physiol.

[CR6] Fiskejo G (1990). Ocurrence and degeneration of “Al-structures” in root cap cells of *Allium cepa* L. after Al-treatmente. Hereditas.

[CR7] Gupta N, Gaurav SG, Kumar A (2013). Molecular basis of aluminium toxicity in plants: a review. Am J Plant Sci.

[CR8] He H, Zhan J, He L, Gu M (2012). Nitric oxide signaling in aluminum stress in plants. Protoplasma.

[CR9] Ishikawa S, Wagatsuma T, Takano T, Tawaraya K, Oomata K (2001). The plasma membrane intactness of root-tip cells is primary factor for Al-tolerance in cultivars of five species. Soil Sci Plant Nutr.

[CR10] Katsuhara M, Kawasaki T (1996). Salt stress induced nuclear and DNA degradation in meristematic cells of barley roots. Plant Cell Physiol.

[CR11] Klug B, Horst WJ (2010). Oxalate exudation into the root-tip water free space confers protection from Al toxicity and allows Al accumulation in the symplast in buckwheat (*Fagopyrum esculentum*). New Phytol.

[CR12] Kochian LV, Piñeros MA, Hoekenga OA (2005). The physiology, genetics and molecular biology of plant aluminum resistance and toxicity. Plant Soil.

[CR13] Larsen PB, Degenhardt J, Tai CY, Stenzler LM, Howell SH, Kochian LV (1998). Aluminum-resistant arabidopsis mutants that exhibit altered patterns of aluminum accumulation and organic acid release from roots. Plant Physiol.

[CR14] Li Z, Xing D (2010). Mitochondrial pathway leading to programed cell death induced by aluminum phytotoxicity in *Arabidopsis*. Plant Signal Behav.

[CR15] Liu Q, Yang JL, He LS, Li YY, Zheng SJ (2008). Effect of aluminum on cell wall, plasma membrane, antioxidants and root elongation in triticale. Biol Plant.

[CR16] Liu J, Piñeros MA, Kochian LV (2014). The role of aluminum sensing and signaling in plant aluminum resistance. J Integr Plant Biol.

[CR17] Ma JF, Zheng SJ, Hiradate S, Matsumoto H (1997). Detoxifying aluminum with buck wheat. Nature.

[CR18] Mariano ED, Keltjens WG (2005). Long-term effects of aluminum exposure on nutrient uptake by maize genotypes differing in aluminum resistance. J Plant Nutr.

[CR19] Marquès L, Cossegal M, Bodin S, Czernic P, Lebrun M (2004). Heavy metal specificity of cellular tolerance in two hyperaccumulating plants, *Arabidopsis halleri* and *Thlaspi caerulescens*. New Phytol.

[CR20] Martinez-Estevez M, Muñoz-Sanchez JA, Loyola-Vargas VM, Hernandez-Sotomayor SMT (2001). Modification of the culture medium to produce aluminum toxicity in cell suspensions of coffee (*Coffea arabica* L.). Plant Cell Rep.

[CR21] Matsumoto H, Hirasawa E, Torikai H, Takahoshi E (1976). Localization of absorbed aluminum in pea root and its binding to nucleic acids. Plant Cell Physiol.

[CR22] Minocha R, Minocha SC, Long SL, Shortle WC (1992). Effects of aluminium on DNA synthesis, cellular polyamines, polyamine biosynthetic enzymes and inor-ganic ions in cell suspension cultures of a woody plant, *Catharanthus roseus*. Physiol Plant.

[CR23] Mizuhiro M, Kenichi Y, Ito K, Kadowaki S, Ohashi H, Mii M (2001). Plant regeneration from cell suspension-derived protoplasts of *Primula malacoides* and *Primula obconica*. Plant Sci.

[CR24] Murashige T, Skoog F (1962). A revised medium for rapid growth and bio assays with tobacco tissue cultures. Physiol Plant.

[CR25] Panda SK, Matsumoto H (2007). Molecular physiology of aluminum toxicity and tolerance in plants. Bot Rev.

[CR26] Poot-Poot W, Hernandez-Sotomayor SMT (2011). Aluminum stress and its role in the phospholipid signaling pathway in plants and possible biotechnological applications. UBMB Life.

[CR27] Ramírez-Benítez JE, Chee-González L, Hernández-Sotomayor SMT (2008). Aluminium induces changes in organic acids metabolism in *Coffea arabica* suspension cells with differential Al-tolerance. J Inorg Biochem.

[CR28] Ramírez-Benítez JE, Hernández-Sotomayor SMT, Muñoz-Sánchez JA (2009). The location of aluminium in protoplasts and suspension cells taken from *Coffea arabica* L. with different tolerance of Al. J Inorg Biochem.

[CR29] Rao IM, Miles JW, Beebe SE, Horst WJ (2016). Root adaptations to soils with low fertility an aluminium toxicity. Ann Bot.

[CR30] Rengel Z, Zhang WH (2003). Role of dynamics of intracellular calcium in aluminium-toxicity syndrome. New Phytol.

[CR31] Rueda A, Rojas M, Lobo M, Urrea A, Restrepo C, Botero C, Pelaez C (2011). Stress responses of tomato protoplasts to copper and paraquat. Trop Plant Pathol.

[CR32] Sasaki T, Yamamoto Y, Ezaki B, Katsuhara M, Ahn SJ, Ryan PT, Delhaize E, Matsumoto H (2004). A wheat gene encoding an aluminum-activated malate transporter. Plant J.

[CR33] Sasaki T, Mori IC, Furuichi T, Munemasa S, Toyooka K, Matsuoka K, Murta Y, Yamamoto Y (2010). Closing plant stomata requires a homolog of an aluminum-activated malate transporter. Plant Cell Physiol.

[CR34] Saxena S, Nouri-Aria KT, Anderson MG, Williams R, Eddleston AL (1985). In vitro alpha-interferon treatment of peripheral blood mononuclear cells improves interleukin-2 activity in HBV-related chronic liver disease. J Hepatol.

[CR35] Siddiqui K, Fan KO, Diffley FX (2013). Regulating DNA replication in eukarya. Cold Spring Harb Perspect Biol.

[CR36] Sónia S (2012). Aluminium toxicity targets in plants. J Bot.

[CR37] Surapu V, Ediga A, Meriga B (2014). Salicylic acid alleviates aluminum toxicity in tomato seedlings (*Lycopersicum esculentum* Mill.) through activation of antioxidant defense system and proline biosynthesis. Adv Biosci Biotechnol.

[CR38] Tabuchi A, Matsumoto H (2001). Changes in cell-wall properties of wheat (*Triticum aestivum*) roots during aluminum induced growth inhibition. Physiol Plant.

[CR39] Wagatsuma T, Ishikawa S, Obata H, Tawaraya K, Katohda S (1995). Plasma membrane of younger an outer cells is the primary specific site for aluminium toxicity in roots. Plant Soil.

[CR40] Wallace SU, Anderson IC (1984). Aluminum toxicity and DNA synthesis in wheat roots. Agron J.

[CR41] Wang M, Oppedijk BJ, Lu X, Van Duijn B, Schilperoort RA (1996). Apoptosis in barley aleurone during germination and its inhibition by abscisic acid. Plant Mol Biol.

[CR42] Yamamoto Y, Kobayashi Y, Matsumoto H (2001). Lipid peroxidation is an early symptom triggered by aluminum, but not the primary cause of elongation inhibition in pea roots. Plant Physiol.

[CR43] Young-Sang L, Mitiku G, Anton GE (2001). Short-term effects of Al^3+^ on the osmotic behavior of red beet (*Beta vulgaris* L.) protoplasts. Plant Soil.

[CR44] Zhan J, He HY, Wang TJ, Wang AQ, Li CZ, He LF (2013). Aluminum-induced programmed cell death promoted by AhSAG, a senescence-associated gene in *Arachis hypoganea* L.. Plant Sci.

[CR45] Zheng SJ, Yang JL (2005). Target sites of aluminum phytotoxicity. Biol Plant.

